# Validating an alternate version of the chewing function questionnaire in partially dentate patients

**DOI:** 10.1186/1472-6831-9-9

**Published:** 2009-03-16

**Authors:** Kazuyoshi Baba, Mike T John, Mika Inukai, Kumiko Aridome, Yoshimasa Igarahsi

**Affiliations:** 1 Department of Prosthodontics, School of Dentistry, Showa University, Tokyo, Japan; 2 Department of Diagnostic and Biological Sciences, School of Dentistry, University of Minnesota, Minneapolis, USA; 3 Division of Epidemiology and Community Health, School of Public Health, University of Minnesota, Minneapolis, USA; 4 Removable Partial Denture Prosthodontics, Department of Masticatory Functional Rehabilitation, Tokyo Medical and Dental University, Tokyo, Japan

## Abstract

**Background:**

The aim of this study was to investigate the dimensionality, reliability, and validity of an alternate version of the chewing function questionnaire in partially dentate patients in Japan.

**Methods:**

Subjects were partially dentate patients who attended the prosthodontic clinic at Tokyo Medical and Dental University (N = 491, 71% women, mean age (± SD): 63.0 ± 11.5 years). The questionnaire asked each subject to rate his or her ability to chew 20 common Japanese foods. For each individual, responses were combined to yield a chewing function summary score, with higher scores indicating better self-reported chewing ability. We used exploratory factor analysis to investigate the scores' dimensionality. For validity assessment, we computed the correlations between the chewing function score and oral health-related quality of life (OHRQoL, as measured by the Japanese 14-item Oral Health Impact Profile (OHIP-14)) Internal consistency of scores and test-retest reliability were investigated by asking a subset of subjects (N = 62) to complete the questionnaire twice, 2 weeks apart.

**Results:**

Exploratory factor analysis provided some evidence that self-reported chewing ability can be characterized by a summary score as the original authors suggest. Support for the validity of chewing function scores using the alternate version of the questionnaire was derived from correlations with OHIP-14 scores (r = -0.46, 95% confidence interval (CI): -0.53 to -0.39); thus, better chewing ability was associated with less impaired OHRQoL. Internal consistency was 'satisfactory,' with a Cronbach's alpha of 0.90 (lower limit of 95% CI: 0.89). The test-retest reliability was 'good,' with an intraclass correlation coefficient of 0.69 (95% CI: 0.56 to 0.82).

**Conclusion:**

The alternate version of the chewing function questionnaire can be used as a stand-alone instrument because of the demonstrated reliability and validity of scores obtained using the questionnaire in partially dentate patients.

## Background

The ability to chew is an important component of oral health [1]. In addition, because this ability may affect dietary choices and nutritional intake, it also has consequences for both physical measures of general health [2-5] and perceived general health status, as measured using generic health-related quality of life instruments [6].

Chewing function can be assessed using chewing tests and questionnaires or personal interviews. Whereas chewing tests allow the assessment of masticatory efficiency with some objectivity, questionnaires provide information as to how an individual perceives his or her chewing ability. For many years, most researchers have used the terms 'objective' and 'subjective' when referring to data gathered through laboratory tests and those gathered from patients' self-reports, respectively, implying that the laboratory tests are more valid and that patients' self-reports provide only surrogate information. However, both assessments represent different but complementary information. Slagter et al. [7] and Carlsson and Lindquist [8] reported that patients' ratings of their chewing experience were only weakly related to their ability to chew test foods. Recently, with increasing importance being attached to patient-reported outcomes in dentistry in general [9-11], the patient-reported assessment approach has gained importance for the assessment of chewing ability.

Subjective methods include single-item questions, food lists, and indices to assess chewing ability or eating difficulty. In the case of single-item questions, subjects are asked a simple question about their chewing ability [12-14]. These questions, although easy to answer, are crude measures of chewing ability and do not provide detailed information regarding which foods the subjects had difficulty eating or could not eat at all. Another subjective evaluation method of chewing ability is a food intake questionnaire which categorizes specific foods according to whether they are easy or difficult to bite or chew [9,15-17]. These questionnaires assess the ability to eat or chew a range of foods of varying hardness or textures that were carefully chosen to be most relevant to self-reported chewing ability in each target population. The chewing function questionnaire developed by Sato et al. [16] is one such instrument and has been used in several studies [16,18,19]. Although this questionnaire can be easily completed in a relatively short time and can be administered at the chair side, its validity and reliability have never been thoroughly investigated. Although originally developed for edentulous patients with conventional full dentures, the questionnaire may be useful for other populations, in particular partially dentate patients, as well. In order to assess self-reported chewing ability using this questionnaire across dental populations, successful validations in the new settings are necessary, because dietary habits or food preferences may depend on the population. In addition, the larger item pool of common Japanese foods, from which the current questionnaire items were selected, offers an opportunity to create an alternate version of the instrument. Actually the same authors reported an alternate instrument that used different items that was selected from the same item pool of Japanese foods [20]. Such alternate forms can complement existing instruments in the assessment of the target construct over short time periods when the test-retest effect prevents repeated use of the same instrument [21].

This study aimed to investigate the dimensionality, reliability, and validity of an alternate version of the chewing function questionnaire in partially dentate patients.

## Methods

### Subjects and setting

During the study period (three consecutive weeks in June and July 2007, 507 consecutive partially dentate patients at the prosthodontic clinic of Tokyo Medical and Dental University were invited to participate in this study. Of these, 496 subjects (97.8%) participated in the study and provided written informed consent. We excluded subjects who presented with any acute oral disease or whose general health would interfere with any dental treatment. After these exclusions, data from 491 subjects were analyzed (N = 491, 71% women, mean age (± standard deviation (SD)): 63.0 ± 11.5 years). Mean number of the missing teeth was 9.6 ± 8.2, and 344 subjects (70.1%) were wearing removable dentures. This study was conducted with approval from the ethics committee of Tokyo Medical and Dental University. (Approval number: #135, December 3, 2005)

### Chewing function questionnaire and its item pool

The chewing function questionnaire proposed by Sato et al. [16] asks the subject to rate his or her ability to chew 20 foods selected from 100 common Japanese foods. The same author also proposed an alternate version of the instrument that used different foods selected from the same item pool [20] (Table 1). Subjects were asked whether it was easy ('1') or difficult ('0') to chew the foods. Item responses for each individual were combined to produce a summary score of 0–20 for that subject that was termed the 'chewing function score,' with higher scores indicating better chewing ability. In this study we validated the alternate version of the chewing function questionnaire.

**Table 1:** Foods listed in the alternative version of the chewing function questionnaire, and the proportion of subjects who reported a particular food to be "easy to chew."

Food*	**Percentage who answered "easy" (%)**
TOFU **	99.6
Pudding	99.0
RICE	97.0
UDON Noodle	95.9
Lettuce	90.2
Shrimp TEMPURA	87.0
Cucumber	82.5
Beef steak **	80.9
Baked RICE CAKE **	58.9
PICKLED RADISH **	53.6
Hard Biscuit **	53.4
MILLET and RICE CAKE	50.3
MARINATED OCTOPUS	46.6
Cockle	46.4
Hard PICKLED RADISH **	35.9
Hard RICE CRACKER **	34.4
Chewing gum **	33.0
Whole apple **	25.9
Dried CUTTLEFISH **	23.8
Cutting cotton thread	18.1

*Foods are listed in descending order of the percentage of subjects who reported a particular food to be easy to chew. Uppercase text indicates a Japanese food.

** Food items that were identical to those used in the original questionnaire.

### Assessment of dimensionality, reliability, and validity

We assessed dimensionality by using exploratory factor analysis (EFA). Following the procedure suggested by Woods [22], we subjected a tetrachoric correlation matrix to EFA by using a weighted least squares estimator to obtain factor loadings with the Mplus program, version 5 [23]. We retained factors with eigenvalues > 1 and rotated them with 'quartimin,' which is an oblique rotation method. Items were assigned to retained rotated factors that had a loading of ≥ 0.5 in absolute value [24].

We differentiated uni-versus multi-dimensionality based on the magnitude of factor loadings, distribution of variance among the factors, and correlation among factors. High correlation of all items with the first retained factor, substantial variance attributed to the first retained factor, and substantial correlation among the retained factor favored unidimensionality.

Test-retest reliability and internal consistency analyses were performed to assess reliability. Internal consistency was measured in the whole sample using Cronbach's alpha [25] and was judged according to previously established recommendations [26]. In a convenience subset of the subjects (N = 62), test-retest reliability was assessed by asking subjects to complete the questionnaire twice, 2 weeks apart. Intraclass correlation coefficients (ICCs) were calculated for the chewing function score according to Shrout and Fleiss's ICC using a one-way analysis of variance [27]. The quality of the reliability coefficients was evaluated using previously established guidelines [28].

For validity assessment, we investigated how OHIP items, OHRQoL domains suggested by Slade [29], and the construct as a whole were correlated with perceived chewing ability. We expected a substantial correlation between perceived chewing ability and OHRQoL because the construct as a whole would capture the direct and indirect consequences of chewing problems. In addition, we expected higher correlations for items and domains related to eating and oral function or physical aspects of oral health compared to items/domains not directly related to these aspects of oral health. We computed the Pearson correlation coefficients between the chewing function score and oral health-related quality of life (OHRQoL, as measured using the summary score of the Japanese 14-item Oral Health Impact Profile (OHIP-14)) [30] as well as between the chewing function score and each of the 7 OHIP domain scores. Spearman rank correlation coefficients were computed between the chewing function score and each of the OHIP item responses. In addition, we computed a Pearson correlation coefficient between the chewing function score and the number of teeth. We hypothesized that more teeth would be related to better chewing ability. Data for one subject were excluded from the analysis because a number of missing OHIP items precluded the calculation of an informative summary score. Individual OHIP items were missing in less than 1% of the sample.

Except for factor analysis, all analyses were performed using the statistical software package STATA Release 9 (Stata Statistical Software 2005; StataCorp LP, College Station, TX, USA), with the probability of a type I error set at the 0.05 α level.

## Results

### Frequency of self-reported ability to eat typical foods

Four foods (tofu, pudding, rice, and udon noodles) were rated 'easy to chew' by almost all (> 95%) subjects (Table 1). Only a third or fewer subjects found (cutting) cotton thread, dried cuttlefish, whole apple, or chewing gum 'easy to chew.'

### Chewing function scores in all subjects and subgroups

The mean (SD) chewing function score for all subjects was 12.1 (4.8) units. When the study population was subdivided into 2 groups at the median age (65 years), younger subjects (n = 237) demonstrated slightly better chewing ability than older subjects (n = 254; 12.5 ± 5.1 versus 11.8 ± 4.6 units, respectively). A comparison of the chewing function scores of female and male subjects indicated a slightly lower chewing ability for female than for male subjects (12.0 ± 4.8 units, n = 350 versus 12.4 ± 4.8 units, n = 141, respectively).

### Dimensionality of chewing function questionnaire

All items correlated substantially with the latent factor (all loadings ≥ 0.39) when only one factor was retained (eigenvalue: 12.1) in the factor analysis. This factor explained 60% of the variance (Table 2). When the eigenvalue criterion of > 1 was applied, 2 factors were retained, with the second having an eigenvalue of 2.8. Together, the 2 factors explained 74% of the variance. When only the item loadings with correlations ≥ 0.5 with the rotated factors were considered important, a clear and simple structure emerged. Four items loaded on the first factor, and the remaining items loaded on the second factor. The 2 latent factors correlated with r_Pearson _= 0.25. The 2 factors were termed 'foods very easy to chew' and 'foods not so easy to chew.'

**Table 2:** Factor loadings for the chewing function questionnaire from the 1- and 2-factor solution (weighted least squares with quartimin rotation).

**Food**	**Factor loadings shown when > 0.5**		
	**1-factor**	**2-factor**	
		
		**1**	**2**
TOFU	0.39	1.12*	
RICE	0.64	0.56	
UDON Noodle	0.69	0.55	
Pudding	0.40	0.74	
Lettuce	0.71		0.56
Shrimp TEMPURA	0.78		0.62
Cucumber	0.84		0.71
Baked RICE CAKE	0.76		0.76
Beef steak	0.79		0.75
PICKLED RADISH	0.95		0.86
MARINATED OCTOPUS	0.90		0.83
Hard Biscuit	0.85		0.91
MILLET and RICE CAKE	0.89		0.93
Hard RICE CRACKER	0.89		0.96
Cockle	0.89		0.87
Hard PICKLED RADISH	0.94		0.91
Dried CUTTLEFISH	0.92		0.96
Chewing gum	0.67		0.72
Whole apple	0.75		0.80
Cutting cotton thread	0.70		0.77

Only correlations of ≥ 0.5 are shown. Uppercase text indicates a Japanese food.

* For rotated solutions, the loadings might be slightly less than -1 or slightly greater than +1, because the factors are not orthogonal with an oblique rotation.

### Reliability

Internal consistency reached a 'satisfactory' level with a Cronbach's alpha of 0.90 (lower limit of 95% confidence interval (CI): 0.89) for all items. Cronbach's alpha was 0.91 (lower limit of 95% CI: 0.90) for the first factor and 0.54 (lower limit of 95% CI: 0.48) for the second factor.

Test-retest reliability was ICC = 0.69 (95% CI: 0.56–0.82) for the chewing function score. This level of reproducibility was considered 'fair to good' and almost reached the 0.70 threshold for 'excellent' reliability.

### Validity

All observed associations among self-reported chewing ability, oral health-related quality of life, and number of teeth were in agreement with the hypotheses. The Pearson correlation coefficient between the chewing function score obtained using the alternate version of the questionnaire and the OHIP-14 summary score was r = -0.46 (N = 490, 95% CI: -0.53 to -0.39), indicating that subjects with higher chewing function scores (which reflect better self-reported chewing ability) had lower OHIP scores (which reflect less impaired OHRQoL). Therefore, better self-reported chewing ability was correlated with better OHRQoL.

Pearson correlation coefficients between the chewing function score and the seven OHIP domain scores ranged from -0.24 to -0.44 with the highest absolute value observed for the domain 'Physical Disability'. The domain 'Functional Limitation' correlated in absolute magnitude with chewing function scores only slightly lower with -0.42 as well as the domain Physical Pain with -0.43. Spearman rank correlations between the chewing function score and the OHIP item responses ranged from -0.17 (irritable with other people) to -0.43 (uncomfortable to eat). All correlation coefficients were statistically significant (p < 0.001).

In addition, the Pearson correlation coefficient between the chewing function score and the number of teeth was r = 0.34 (N = 491, 95% CI: 0.26 to 0.41), indicating that a greater number of teeth was associated better self-reported chewing ability.

## Discussion

This study was designed to investigate the dimensionality, reliability, and validity of an alternate version of the chewing function questionnaire [16] in partially dentate patients.

When we investigated the dimensionality of the alternate version of the chewing function questionnaire, EFA revealed the existence of 2 factors. The first factor contained 16 items, while the second contained 4 items. The second factor was characterized by items with very high prevalence, as eating tofu, rice, udon, and pudding was possible for > 95% of our subjects. However, because of the strong first factor, the substantial correlation of all items with this latent variable, and the low prevalence of the items related to the second factor, we considered perceived chewing ability as a construct that could be characterized by a summary score. Although we found a second factor that contained foods that were rated easy to chew by almost all of our partially dentate subjects and could therefore have been deleted because they did not provide much information about our subjects, we think that these items may be useful in other patient populations with lower chewing ability, e.g., patients with TMD-related pain. In such populations, patients may have more difficulty to chew these foods, and the items would therefore provide information for the discrimination of patients. To maintain the comparability of the chewing function scores, retaining these 4 items may be advised even for partially dentate patients. In addition, the original Sato questionnaire also considered self-reported chewing ability a uni-dimensional construct because only one summary score is formed, which is in line with other reports that considered their instruments as characterizing a single construct [6,31]. However, we believe that our EFA provided an initial insight into the dimensionality of perceived chewing ability. We used an exploratory technique because we considered this the appropriate step for an evaluation of a construct where factor analytic techniques have not been applied before and only expert opinion of the construct structure was available. In this situation, EFA is often recommended as the first analytic approach [32]. According to our findings, dimensions of perceived chewing ability may exist. This hypothesis could be tested using confirmatory factor analysis (CFA) against alternative models of perceived chewing ability, in particular, a uni-dimensional model. Future research involving CFA-related multi-variable statistical techniques such as structural equation modelling as well as qualitative analyses [33] may provide further insights into the structure of perceived chewing ability

We consider the results sufficient to justify the instrument's use to discriminate subjects with different levels of perceived chewing ability in a typical target population for the questionnaire. We demonstrated that the chewing function questionnaire can be used in a different population than the one in which it was originally developed. Therefore, this instrument provides an opportunity for evaluating perceived chewing ability in patients with minimal to complete tooth loss. Furthermore, the above results, taken in conjunction with other data that support the utility of this instrument in another population with chewing problems (patients with temporomandibular disorders (TMDs) [19]) suggest that this instrument provides a unified approach to measure perceived chewing ability in the Japanese culture across populations with limited chewing function.

Only limited information is available in the literature regarding the reliability of instruments (questionnaires) for the measurement of perceived chewing ability. In fact, no such data are available for the chewing function score. For the Chewing Ability Index, another instrument for the assessment of chewing ability, the coefficient of reproducibility was reported to be 0.98 [9]. Reproducibility for the Index of Eating Difficulty, an instrument developed in China, was reported to be 0.99, and the weighted kappa for test-retest reliability was reported to be 0.89 [34]. Although direct comparison is not possible due to methodological differences, these previously reported data are in general agreement with our study results. They support the notion that the assessment of perceived chewing ability in general is possible with sufficient reliability. When we investigated internal consistency, the results were rated 'excellent,' according to guidelines, and the test-retest reliability for the chewing function score was slightly lower, but still ranked 'fair to good.' These results suggest that reliability of the alternate version of the chewing function questionnaire is sufficient, with the internal consistency aspects of reliability being better than its test-retest reliability, which was slightly lower than expected.

We utilized an OHRQoL measure because the ability to chew has been reported to be an important dimension of OHRQoL [6] and chewing ability has been associated with oral function related impact. We did not use other established perceived chewing ability index such us the one developed by Leake [9]. This instrument, the Chewing Ability Index [9], is a 0 – 5 score based on self-reported ability to chew 5 foods and it has been reported to be valid as a measure to evaluate perceived chewing ability. However, a validated Japanese version of the instrument was not available, and unfortunately some of food items listed in the questionnaire are not very common in Japan. It is a limitation of our study that we did not incorporate the other chewing function questionnaire. We consider the two instruments interchangeable and very highly correlated because they are derived from the same original 100-item pool, and they share a considerable number of items.

When we investigated the score validity, the observed associations among self-reported chewing ability, oral health-related quality of life, and the number of teeth met a priori expectations. A positive relationship existed between the number of teeth and the chewing function scores, which has been previously observed among older adults [35-37]. Other reports have shown that oral conditions such as infected or sore gums, loose teeth, and toothache – all possible precursors of tooth loss – were associated with the onset of chewing difficulty [38]. Chewing ability should be associated with oral function related impacts. Therefore, the observed associations between chewing ability and OHRQoL, where chewing is part of the 'oral function' dimension [39,40], with the 2 items characterizing the 'physical disability' or 'functional limitation' domain in the OHIP-14, also supported the validity of the questionnaire scores. That the strongest correlation between each item and the chewing function scores was observed for the item 'uncomfortable to eat)' fitted a priori expectations. These findings are in agreement with those of previous studies [6,34] reporting a significant association between chewing ability and OHRQoL.

Based on the sufficient psychometric properties of the alternative version of the chewing function questionnaire the opportunity exist to assess the target construct over very short periods of time when test-retest effects are expected that would prevent assessment with the same instrument. We do not know the magnitude of test-retest effects for perceived chewing ability; however, for a related construct, OHRQoL, the test-retest effects were present but small [21]. Therefore, test-retest effects may also occur with instruments used to measure perceived chewing ability and may motivate the use of the alternate version. In addition, more information relevant to the diagnosis of chewing problems and assessment of treatment outcomes can be obtained using an alternate version together with the original questionnaire.

## Conclusion

The alternate version of the chewing function questionnaire can be used as a stand-alone instrument for perceived chewing ability evaluation because of the demonstrated reliability and validity of scores obtained using the questionnaire in partially dentate patients.

## Competing interests

The authors declare that they have no competing interests.

## Authors' contributions

KB carried out the outcome studies, participated in the sequence alignment and drafted the manuscript. MI carried out the data collection. KA participated in the sequence alignment. MTJ participated in the design of the study and performed the statistical analysis. YI conceived of the study, and participated in its design and coordination. All authors were involved in the manuscript preparation and approved the final manuscript.

## Pre-publication history

The pre-publication history for this paper can be accessed here:


